# An Improved Crucible Spatial Bubble Detection Based on YOLOv5 Fusion Target Tracking

**DOI:** 10.3390/s22176356

**Published:** 2022-08-24

**Authors:** Qian Zhao, Chao Zheng, Wenyue Ma

**Affiliations:** 1 School of Communication and Information Engineering, Xi’an University of Science and Technology, Xi’an 710054, China; 2 Xi’an Dishan Vision Technology Limited Company, Xi’an 712044, China

**Keywords:** YOLOv5, target detection, quartz crucible bubbles, feature fusion, Kalman filter, Hungarian algorithm

## Abstract

A three-dimensional spatial bubble counting method is proposed to solve the problem of the existing crucible bubble detection only being able to perform two-dimensional statistics. First, spatial video images of the transparent layer of the crucible are acquired by a digital microscope, and a quartz crucible bubble dataset is constructed independently. Secondly, to address the problems of poor real-time and the insufficient small-target detection capability of existing methods for quartz crucible bubble detection, rich detailed feature information is retained by reducing the depth of down-sampling in the YOLOv5 network structure. In the neck, the dilated convolution algorithm is used to increase the feature map perceptual field to achieve the extraction of global semantic features; in front of the detection layer, an effective channel attention network (ECA-Net) mechanism is added to improve the capability of expressing significant channel characteristics. Furthermore, a tracking algorithm based on Kalman filtering and Hungarian matching is presented for bubble counting in crucible space. The experimental results demonstrate that the detector algorithm presented in this paper can effectively reduce the missed detection rate of tiny bubbles and increase the average detection precision from 96.27% to 98.76% while reducing weight by half and reaching a speed of 82 FPS. The excellent detector performance improves the tracker’s accuracy significantly, allowing for real-time and high-precision counting of bubbles in quartz crucibles. It is an effective method for detecting crucible spatial bubbles.

## 1. Introduction

Quartz crucibles are widely used in the preparation of solar cells and integrated circuits as a critical material for single-crystal silicon production, and they directly impact the quality of the prepared products [1]. The existing technology produces quartz crucibles with a two-layer structure with a transparent inner layer and an opaque outer layer, the transparency of which is caused by the number and size of bubbles. The outer wall contains many dense bubbles, which give a flocculent opaque appearance, increase thermal insulation, and provide a uniformly radiating heat source. In contrast, the inner wall contains sparse, tiny bubbles, which rise in size during the 50-h high-temperature exposure to 1400 °C and can easily rupture, allowing the gas and quartz impurities in the bubbles to penetrate the silicon solution and destroy the crystal structure [2]. Therefore, before using the crucible, it is crucial to check the size and quantity of bubbles in the transparent layer of the inner wall.

In industry, bubble measurement techniques are widely used. In Ref. [3], a new method is used for measuring the bubble size distributions of 2D highly clustered bubbles using image processing technique. The diameters and size distribution of bubbles can be statistically calculated after binarization, edge extraction, and hole filling of the captured image. In Ref. [4], a machine vision method based on edge pixel-based edge detection and target region locking based on a calibrated connected domain was proposed to detect bubbles in crystals, instead of the human eye. The detection method significantly improved the detection rate and accuracy of identifying bubbles in sapphire and determining their location. However, the traditional algorithm is limited by the lighting environment at the time of imaging and the existence of small bubble targets with less feature information, lower resolution, incomplete boundary contours, and other factors, which have insufficient generalization ability and target miss detection, and cannot meet industrial detection needs [5]. Current deep learning-based target detection algorithms are mainly divided into two-stage and single-stage target detection algorithms, in which the single-stage target detection algorithm has a simple structure and higher computational efficiency [6]. Compared with traditional image processing algorithms, deep learning-based target detection algorithms use the powerful feature extraction ability of convolutional neural networks in a large number of data samples to obtain target information-rich feature maps, effectively solving the difficulties of traditional algorithms. The models are highly modular, and they can be applied to visual measurement and defect detection tasks in industry [7,8], medicine [9], and other fields by improving different structures [10,11]. Specifically, in Ref. [12], the authors realized the detection of bubble defects in tire crown speckle interference based on the Faster R-CNN network framework and redesigned the feature pyramid structure to improve the small target detection precision. Still, many inference calculations reduced the detection speed and did not involve spatial bubble tracking. In order to improve the accuracy of short-term vehicle tracking in the process of autonomous driving, in the work of [13], a method combining YOLOv3 and Kalman filtering was proposed to realize real-time warnings for those objects that were completely blocked. It was more suitable for autonomous driving applications. To address the problem of the slowness of the existing statistics based on mosaic images, in Ref. [14], a method based on the YOLOv3 model and the SORT algorithm was used to perform the statistics of spruce number in a UAV-captured aerial video of complete spruce plots. The method could quickly and accurately calculate the number of spruce in a complete plot. However, the detection algorithms used YOLOv3, which limited the performance of the tracking algorithm.

The detector’s quality significantly impacts the tracking performance [15]. In this paper, to improve the accuracy of the final quantity count, firstly, the network structure is improved based on the current YOLOv5 with better all-around performance, using the dilated convolution to compensate for the missing deep semantic features and enhancing the critical channel feature weights by using an efficient channel attention network. After that, the crucible bubble dataset constructed is trained and validated to improve the accuracy and speed of the network for small bubble detection. Lastly, the Kalman filter and Hungarian algorithm are used to correlate the upper and lower frame data to count the number of bubbles in the crucible’s transparent layer space and provide data references and technical support for quartz crucible quality inspection.

## 2. Related Work

### 2.1. Video Image Acquisition

**Figure 1:**
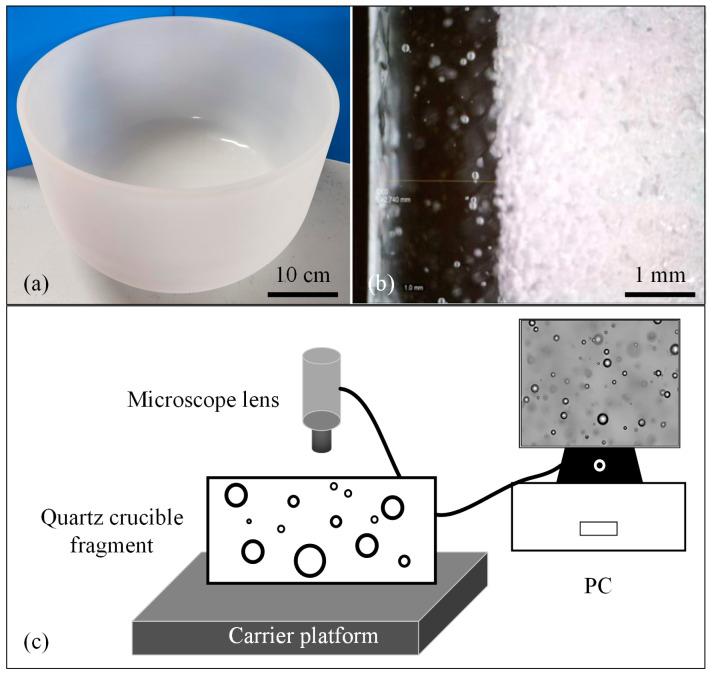
Image acquisition. (**a**) Picture of quartz crucible; (**b**) Double structure inside the quartz crucible; (**c**) Schematic diagram of the acquisition system.

The quartz crucible is white, cup-shaped (as shown in Figure 1a), with a double-layer structure. As shown in Figure 1b, the outer side is an opaque structure with dense and relatively large bubbles, all between 50 and 300 µm in diameter; these bubbles are required for the mechanical stability of the crucible in order to provide a uniformly radiating heat source for the built-in silicon block and enhance the conversion efficiency [2]. The inner side is a 3–5 mm transparent layer, and the presence of trace bubbles can result in the release of particles from the crucible into the melt, which may inhibit single-crystalline growth; most of these conventional bubbles are between 10 and 100 µm in diameter. The video image acquisition system built in the laboratory consists primarily of a host, a camera, a fixed-focus lens, a remote-control handle, and other laboratory components. Figure 1c shows the schematic diagram of the image acquisition system. Because the real size of the bubbles in the quartz crucible is at the micron level, ordinary cameras cannot capture clear bubble images. The VHX7000 series digital microscope from Keyence, Japan, is selected as the acquisition equipment, and it can acquire bubbles as small as 7 microns in diameter. When the magnification is approximately 200, clear bubbles can be observed visually. A remote-control handle can also be used to control the vertical movement of the carrier stage up and down in the Z-axis direction, allowing images of bubbles at different depths to be acquired by varying the object distance. Quartz crucible fragments are used as image acquisition objects to facilitate video acquisition.

**Figure 2:**
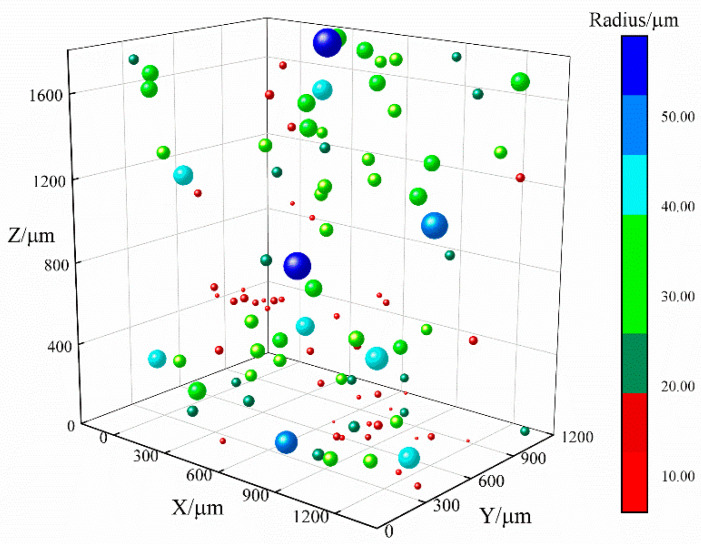
Schematic diagram of the bubble distribution in the crucible space.

**Figure 3:**
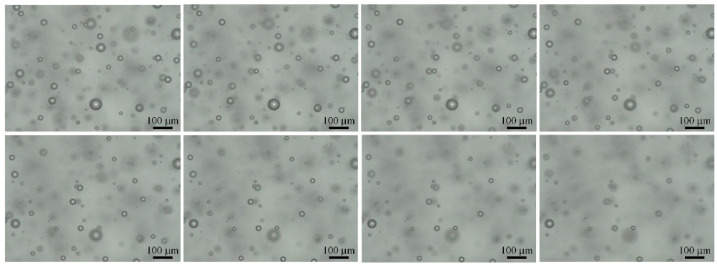
Bubble image change in continuous frames.

In order to obtain bubble images of different depths, it is necessary to constantly adjust the object distance of the microscope. During the movement of the carrier platform, images of bubbles with varying depths were captured on video. According to the optical imaging principle of the microscope vision system, when the center of the bubble sphere is on the focal plane of the digital microscope, the edge of the bubble is clear and sharp, with a low grey value, indicating a focused bubble. When the center of the bubble sphere is slightly off the focal plane of the digital microscope, the edge of the bubble is blurry, indicating an unfocused bubble, and when the center of the bubble sphere is completely outside the focal plane of the digital microscope, the bubble disappears from the image. Therefore, the feature view of bubble appearance changes constantly in the captured video images. Moreover, due to the movement of the carrier platform, video jitter will inevitably occur, and the bubble position will change slightly. Figure 2 shows a schematic diagram of the three-dimensional bubble distribution in the crucible space. Figure 3 shows the captured video image of the bubble variation segment.

### 2.2. Principle of YOLOv5 Algorithm 

**Figure 4:**
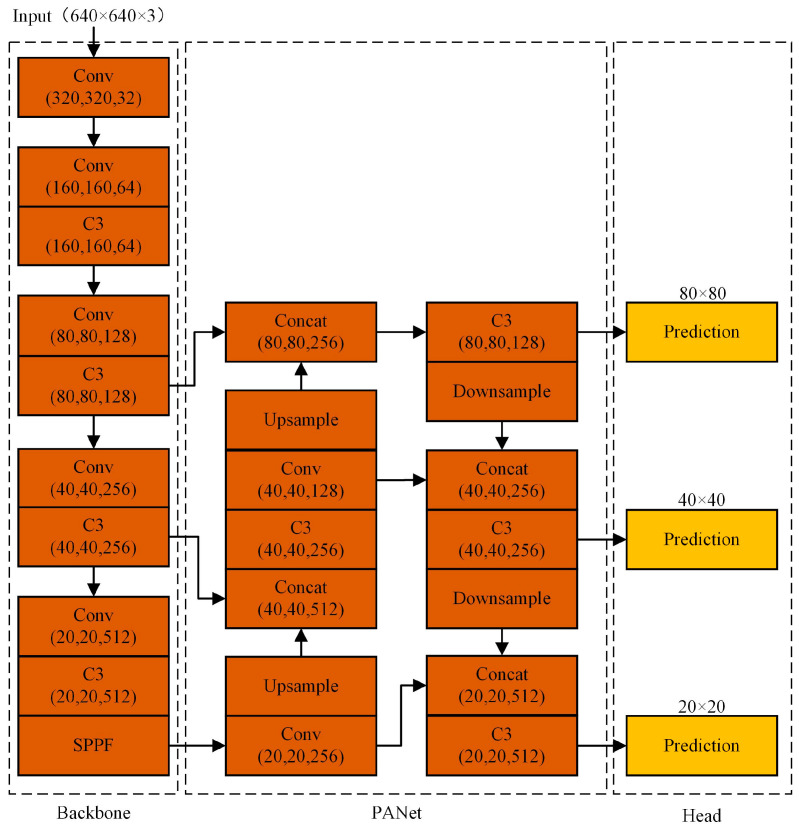
Structure of YOLOv5s network.

The YOLO series combines speed and precision with a more robust capacity for generalization. YOLOv5 achieves state-of-the-art performance, and the algorithm is currently available in a variety of sizes, including YOLOv5s, YOLOv5m, YOLOv5l, YOLOv5x, etc. As the network with the smallest width and depth, YOLOv5s meets the requirements for industrial detection precision while incorporating the advantages of embedded deployment speed and simplicity. Its network architecture is shown in Figure 4.

**Figure 5:**
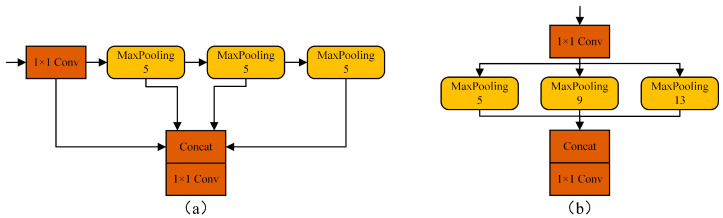
Muti-sensory filed fusion structure. (**a**) SPPF serial pooling structure; (**b**) SPP parallel pooling structure.

Backbone network: using a 640 × 640 input image as an example, the backbone network continuously down-samples the image to obtain the feature map, then uses the C3 module for feature extraction to obtain deep features, and finally uses the SPPF module to incorporate the crucial contextual information. The C3 module borrows the idea of a lightweight CSP-Net [16] structure, which can reduce network computation, realize the fusion of rich gradient information, and improve network learning capability. Spatial Pyramid Pooling Fast (SPPF) is an improved version of Spatial Pyramid Pooling (SPP). The improved SPPF is a serial pooling structure with three pooling kernels of size 5, which can effectively utilize the pooling output of the upper layer and achieve the fusion of multiple perceptual field features at a lower computational cost than the parallel pooling structure, with kernels of size 5, 9, and 13 utilized by SPP. The structure of SPPF is shown in Figure 5a, while the structure of SPP is shown in Figure 5b.

Neck network: the three scale feature maps of 80 × 80, 40 × 40, and 20 × 20 obtained by 8, 16, and 32 times down-sampling of the backbone network are fused by a Path Aggregation Network (PANet) [17] for shallow and deep feature fusion. This extracts the semantic information of the image’s deeper features while fusing the detailed information of the image’s shallow features.

Head detection: due to the high resolution of the shallow feature map, the predicted target box size is small and densely distributed, enabling the detection of small targets; the middle feature and deep feature maps have a lower resolution, and the predicted target box size is larger and sparsely distributed, enabling the detection of medium and large targets. For each position of each scale feature map, the head network predicts three target boxes. If the number of categories predicted by the network is k, then 5 + k prediction values are calculated for each target box, where the first four values are used to adjust the target box’s position and the fifth value indicates the confidence level that the target box contains a target.

## 3. Method

### 3.1. Detector

#### 3.1.1. Improved YOLOv5-QCB Network Structure

**Figure 6:**
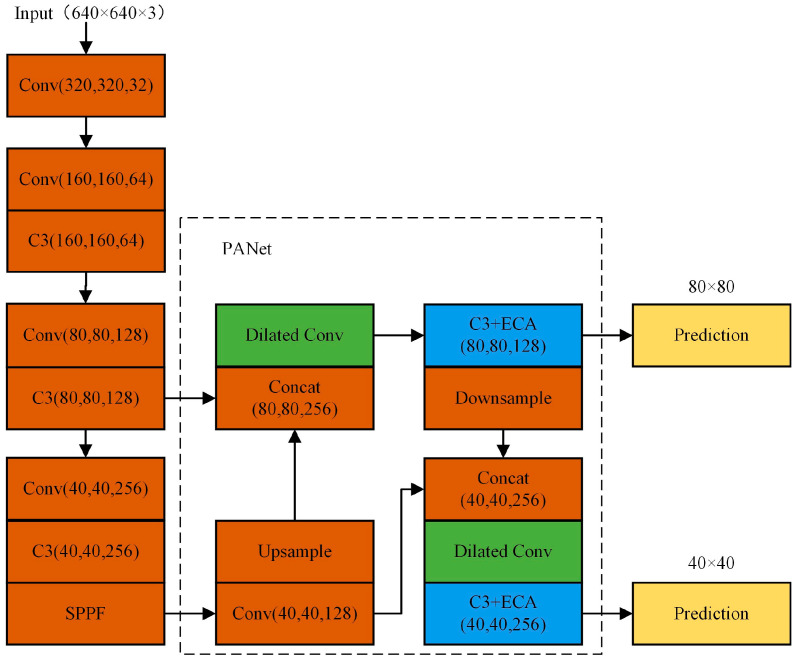
Proposed YOLOv5-QCB structure in this paper.

The recall of target detection and the precision of the detection box are important for the subsequent target tracking; therefore, in this section, an efficient and feasible bubble detection method is proposed through relevant strategies and experiments, yielding optimal final bubble number statistics. As a general framework for locating targets, the current YOLOv5 network model is effective at both locating and identifying targets. Nonetheless, the detection performance is not optimal due to the poor image illumination under the microscope and the blurred edge contours of small bubbles. The improved YOLOv5-QCB bubble detection network is proposed in this paper, and its overall structure is depicted in Figure 6.

First, for the small and dense size of the dataset bubble targets, the relevant feature layers in the backbone network with 32-fold down-sampling are deleted. The shallow feature map with 8-fold down-sampling (80, 80, 128) and the medium feature map with 16-fold down-sampling (40, 40, 128) are fused with feature information through PANet. After that, the head uses two scale feature maps of 40 × 40 and 80 × 80 to detect medium-size and small-size targets, respectively.

Second, because the deletion of the 32-fold down-sampled feature layer will deprive the network of certain deep semantic features, the global semantic features are obtained by expanding the convolutional kernel perceptual field using dilated convolution in the PANet structure.

Lastly, because the original head detection network treats all channels equally in a seemingly fair but unreasonable way, it is vital to enhance the detailed feature information of small bubbles for the task of small bubble detection. Therefore, prior to head detection, the ECA-Net mechanism is used to strengthen the important channel feature weights to make the network pay more attention to the task-relevant channel features.

#### 3.1.2. K-Means Clustering Anchor Box

To improve the network target box regression capability, the K-means clustering algorithm was used to re-cluster this dataset to obtain six prior anchor box sizes for the combined theory and assign them to the output feature maps at both scales by size (as shown in Table 1).

According to the definition of target size by absolute size in the COCO dataset, targets smaller than 32 × 32 in size are classified as small, those larger than 96 × 96 are classified as large, and those between 32 × 32 and 96 × 96 are classified as medium. According to the clustered anchor box sizes, there are no large-size targets in this dataset, and the width-to-height ration of the majority of anchor boxes is 1:1, which is consistent with the characteristics of crucible transparent layer bubbles.

#### 3.1.3. Dilated Convolution

**Figure 7:**
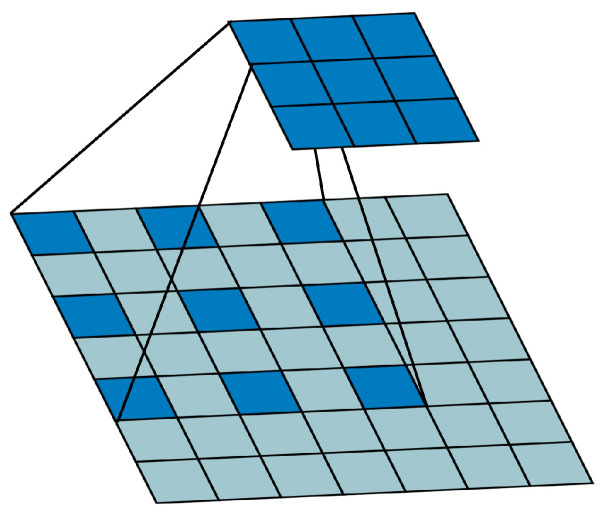
Illustration of dilated convolution.

As the layers of the neural network deepen, the feature map perceptual field is generally enlarged by down-sampling and increasing the size of the convolutional kernel. However, down-sampling causes the feature map resolution to decrease and detailed information to be lost, whereas increasing the size of the convolutional kernel introduce a large number of parameters and computation and reduces the network performance. Dilated convolution is to zero-fill the convolution kernel of standard convolution to expand the perceptual field without increasing the computational effort, while maintaining the resolution of the output feature map and avoiding the problem of loss of detailed information due to down-sampling, which is more effective for the detection of small targets [18]. In this paper, to compensate for the semantic features lost by removing the deep feature map, the feature map perceptual field is expanded using dilated convolution with a kernel size of 3 × 3, a step size of 1, and an inflation rate of 2 (as shown in Figure 7).

#### 3.1.4. Introduction of ECA-Net Mechanism

**Figure 8:**
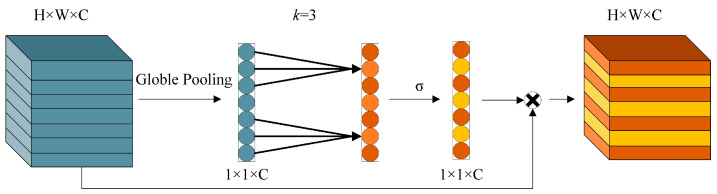
Structure of the ECA module.

Because the detection objects in this paper are small-sized targets with limited feature information and the original network as a generic model lacks an optimization strategy for small targets, there is a problem with insufficient feature information extraction capability. On the other hand, the attention mechanism improves the detection performance by enhancing the important feature information, with almost no increase in model size and computational effort. The earliest proposed Squeeze-and-Excitation (SE) [19] module is widely implemented. Still, dimensionality reduction inevitably results in the loss of feature information, and computing the dependencies among all channels is inefficient and unnecessary. Unlike the SE module, the ECA [20] module does not reduce computation by dimensionality reduction. Instead, after global averaging pooling of the input feature map to obtain a 1 × 1 × C feature map with the global perceptual field, fast one-dimensional convolution with a kernel size of *k* is implemented for each channel and its *k* nearest neighbors via local cross-channel information interaction. The size of the convolution kernel, *k*, is proportional to the number of input channels, and the local channel dependencies are obtained by compressing the channel weights between 0 and 1 using the Sigmoid function. This enables efficient channel feature value weighting and draws the network’s attention to task-related channel feature information. The structure of the ECA module is shown in Figure 8.

### 3.2. Tracker

#### 3.2.1. SORT Target Tracking

**Figure 9:**
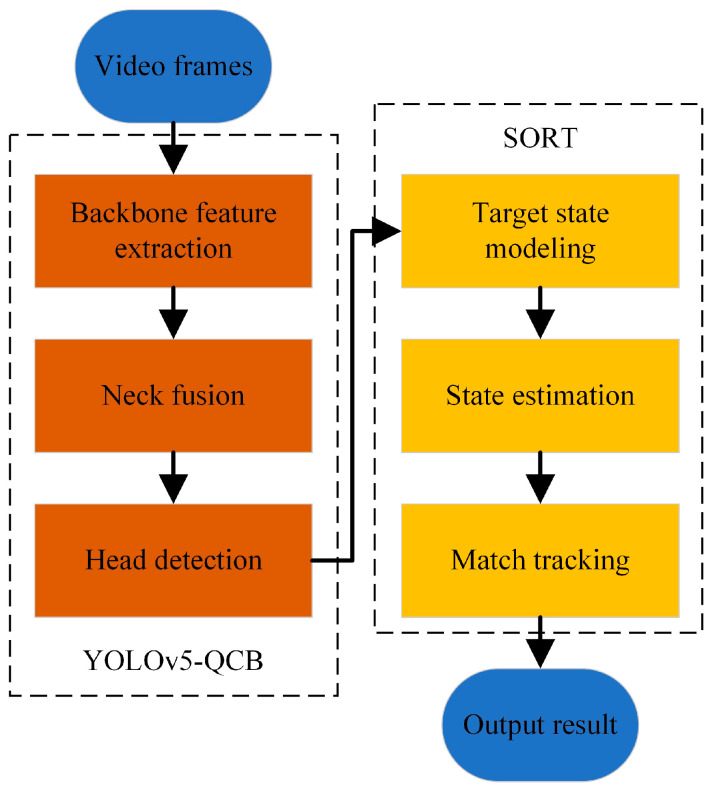
Flow chart of the tracking algorithm.

The target tracking algorithm SORT has the advantages of fast speed, low arithmetic power consumption, and simple computation, and it has been extensively used in vehicle and pedestrian tracking and number counting. In this section, to solve the problem of monitoring and counting bubbles in the transparent layer space of the crucible under the video, joint motion estimation and adjacent inter-frame matching target tracking algorithms are proposed based on the improved bubble detection network. Firstly, the video frames are input into the improved YOLOv5-QCB target detection network, and the output target box information is obtained by backbone network feature extraction and neck feature fusion. This is then input into the target tracking framework. Because the bubble appearance feature view changes continuously by constantly adjusting the object distance to obtain bubble images of varying depths, the position and size of the target box are used to model the bubble state, followed by state estimation and association to match upper and lower frame targets (as shown in Figure 9).

#### 3.2.2. Bubble State Modeling

In this paper, bubble tracking based on the SORT algorithm is divided into three stages: modeling bubble state, estimating motion state, and associating data. Initially, we model the motion estimation based on the position and size of the bubble detection box and define a six-dimensional state vector to represent the bubble’s state information. It is described as follows:

(1) $$X={\left[l_{x},l_{y},w,h,v_{x},v_{y}\right]}^{T}$$

where *l_x_* and *l_y_* are the horizontal and vertical coordinates of the center of the detection box, *w* and *h* are the width and height of the detection box, *v_x_* and *v_y_* are the velocity components of the target along the two axes, and 0 is the initial value.

The bubble’s state estimation is implemented using the Kalman filter [21]. The prediction stage concludes the prediction of the current frame’s target position based on the previous frame’s detection target state data. The bubble state prediction is shown as follows:

(2) $$\left\{ \begin{array}{l} X_{t}=AX_{t-1}\\ P_{t}=AP_{t-1}A^{T}+Q \end{array}\right.$$

where *X_t_* represents the predicted bubble state at frame *t*, *A* represents the state transfer matrix, *X_t−_*_1_ represents the bubble state at frame *t* − 1, *P_t_* represents the error covariance matrix at frame *t*, *P_t−_*_1_ presents the updated error covariance matrix at frame *t* − 1, and *Q* represents the process noise. In the initialization phase of the Kalman filter, the state transfer matrix, *A*, is assigned the following values:

(3) $$A=[\begin{array}{cccccc} 1 & 0 & 0 & 0 & 1 & 0\\ 0 & 1 & 0 & 0 & 0 & 1\\ 0 & 0 & 1 & 0 & 0 & 0\\ 0 & 0 & 0 & 1 & 0 & 0\\ 0 & 0 & 0 & 0 & 1 & 0\\ 0 & 0 & 0 & 0 & 0 & 1 \end{array}]$$

#### 3.2.3. Data Association of Upper and Lower Frames

Establishing a one-to-one correspondence between the tracking target and the detection target is critical for detection-based multi-target tracking. The Hungarian algorithm [22] is utilized in this paper to match the association between the bubble targets of the upper and lower frames. The Hungarian algorithm is a combinatorial optimization algorithm for the assignment problem under the assumption that the current bubble target position predicted in the previous frame is *T*= {*t*_1_, *t*_2_, …, *t_n_*} and the bubble target position detected in the current frame is *D* = {*d*_1_, *d*_2_, …, *d_m_*}. Calculating the intersection ratio between the tracking and detection boxes yields the cost matrix of *m × n*. Following are the formulas for calculating the intersection ratio, *iou*, and the cost matrix, *P*:

(4) $$iou_{ij}=\frac{S_{\mathrm{in}}}{S_{i}+S_{j}-S_{\mathrm{in}}}$$

(5) $$P=\left[\begin{array}{ccc} iou_{11} & \cdots & iou_{1n}\\ \vdots & iou_{ij} & \vdots \\ iou_{m1} & \cdots & iou_{mn} \end{array}\right]$$

where *S_in_* is the area of the overlap region between the two rectangular boxes, *S_i_* denotes the rectangular area of the *i*-th target frame, *S_j_* is the area of the *j*-th target rectangular frame, and *iou_ij_* is the overlap ratio between the *i*-th detection target and the *j*-th tracking target. Finding the solution that minimizes the distance between all tracking and detection boxes yields the optimal matching result.This is because the intersection ratio demonstrates the spatial relationship between the tracking and detection targets.

## 4. Experiments

### 4.1. Experimental Environment and Datasets

This paper’s experiments were conducted on a Windows 10 operating system with a 2.30 GHz Intel Core i7-11800H processor, 16 GB of RAM, an NVIDIA RTX 3050 graphics card with 4 GB of video memory, and CUDA11.1 and CUDNN8.1 supporting GPU acceleration. The input size of the model is 640 × 640, all network parameters are initialized randomly, and 200 cumulative epochs are calculated.

Frame extraction of the collected video data yielded a large number of bubble images, from which 500 were selected as the quartz crucible bubble dataset and manually labeled using LabelImg. This dataset contains a total of 6217 bubble targets, with the labeling category name quartz crucible bubbles (QCB). The manually labeled XML file format was programmed to read each image’s location, target label coordinates, and category information, and it was then converted to txt format so that YOLOv5 could read the dataset. Randomly, the bubble dataset was split into training and validation sets, with a ratio of 8:2. The training set contains 400 images with 4878 bubble target instances, while the validation set contains 100 images with 1339 bubble target instances (as shown in Table 2).

### 4.2. Evaluation Metrics

Typically, Precision (*P*), Recall (*R*), Average Precision (*AP*), mean *AP* (mAP), weight size, and Frames Per Second (FPS) are selected as model evaluation metrics for the evaluation of target detection performance. Precision of detection is computed as shown in Equation (6). If the value is 100%, then there are no false positives. Recall is computed as depicted in Equation (7). If the value is 100%, it indicates that no detections were missed. While the single precision and recall cannot fully evaluate the performance of the network algorithm, the average precision integrates the precision and recall, which is one of the most important performance evaluation indicators of the target detection algorithm, and the formula is shown in Equation (8); mAP represents the average of *AP* across all categories, and when the detection is limited to a single category, mAP is equivalent to *AP*. The size of the weight is used as a measure of model complexity. The lighter the model, the lighter the weight. FPS refers to the number of image frames per second processed by the network and is used to determine the network’s detection speed.

(6) $$P=\frac{TP}{TP+FP}$$

(7) $$R=\frac{TP}{TP+FN}$$

(8) $$AP=\int_{0}^{1}P\left(R\right)dR$$

where *TP* represents the number of positive samples detected correctly, indicating that positive samples were detected as positive samples, *FP* represents the number of positive samples detected incorrectly, indicating that negative samples were detected as positive samples, and *FN* denotes the number of negative samples detected incorrectly, indicating that negative samples were detected as positive samples. *AP* is the area under the *P*-*R* curve, which denotes the average of all the detection accuracies at each recall level.

### 4.3. The Impact of Fusion of Each Depth Feature on Performance

**Figure 10:**
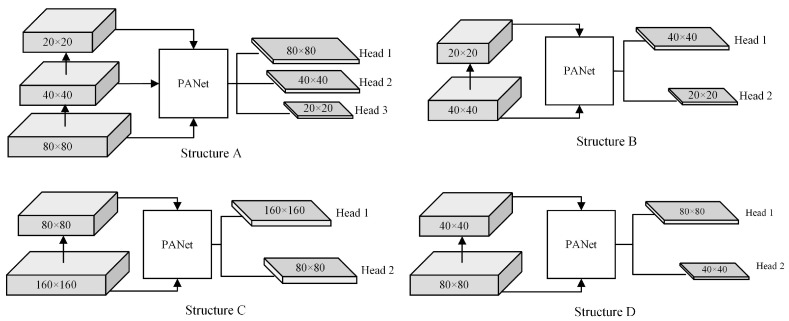
Structures of different depth feature fusion.

The original neck network is comprised of a feature fusion of three different depth feature maps after 8×, 16×, and 32× down-sampling, which is ideal for detecting large and medium-sized targets. For this dataset, excessively large down-sampling multiples will result in the loss of the fine-grained features of small bubble targets; therefore, the effect of each depth feature fusion on the model’s detection performance must be explored. Four fusion structures for the group experiments were selected as follows: A represents the 20 × 20, 40 × 40, 80 × 80 three-scale feature fusion structure of the original network, B represents the 20 × 20, 40 × 40 deep feature fusion structure, C represents the 80 × 80, 160 × 160 high-resolution feature fusion structure, and D represents the 40 × 40, 80 × 80 shallow feature fusion structure. The structures of models A, B, C, and D are shown in Figure 10, and the results are shown in Table 3.

The experiment reveals that structure D has the best performance, with an AP of 97.66%, followed by structure C, structure A, which has poor performance, and structure B, which has the worst performance. This is due to the fact that when the feature layer is shallow, the resolution of the feature map is greater and the detailed information of small targets is more abundant. In contrast, when the feature layer is deepened, the down-sampling operation will acquire deep semantic information, but will lose a substantial amount of detail. Due to the small size of the quartz crucible bubble, structure B, compared to the original structure A, only utilizes the deep semantic information while missing a large number of detailed features, resulting in a significant decrease in the precision of small targets. Compared to structure D, Structure C has more detailed features but fewer semantic features, resulting in a lower detection precision. Compared to structures A and B, structure D is significantly more accurate, reduces file size considerably, and increases detection speed. Although structure D’s detection speed is slower than that of structure C, all other performance indicators are optimal, and the model’s overall performance is more robust. It can be seen that structure D has the best detection performance for small targets because it integrates shallow and deep features more effectively to prevent the loss of detailed features. In this paper, structure D is employed for the detection of bubbles in quartz crucibles, the 32-fold down-sampling layer is eliminated, and two output feature maps with scales of 40 × 40 and 80 × 80 are used for head detection.

### 4.4. YOLOv5-QCB Ablation and Comparison Experiment

**Figure 11:**
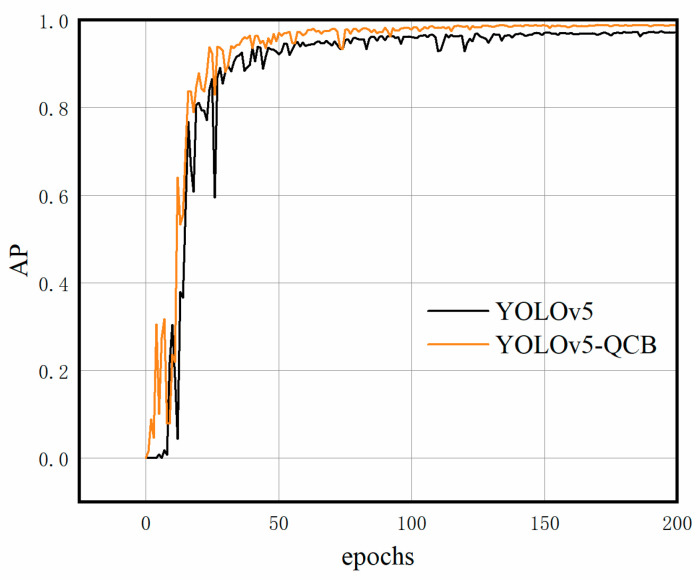
AP curve for network model training.

Figure 11 shows the AP training profiles for the YOLOv5 benchmark network and the improved YOLOv5-QCB model. Both models exhibit a general upward trend, but YOLOv5-QCB has a greater AP value than the original YOLOv5 model. At 110 and 120 iterations, YOLOv5 displays large fluctuations, respectively. At 75 iterations, YOLOv5-QCB displays a significant fluctuation, and it gradually stabilized until convergence. It is evident that YOLOv5-QCB is markedly more stable, and for the same number of iterations, the AP value of the improved YOLOv5-QCB model is better than that of the YOLOv5 model.

In order to demonstrate the efficacy of the improved YOLOv5, the performance improvement effect of the improved shallow network structure, the use of dilated convolution, and the addition of the ECA-Net mechanism are validated through ablation experiments, and the results are presented in Table 4. Model 1 employs a shallow feature network structure, which significantly improves recall and average precision as a result of the deletion of the majority of the deep convolution and greatly reduces the file size while retaining a vast number of shallow detailed features. Model 2, based on the improved shallow network structure, employs dilated convolution to expand the perceptual field of the convolutional kernel and extract more complete and effective global semantic feature information than model 1, thereby improving the model’s detection precision and recall rate. Model 3 is the newly-introduced ECA-Net mechanism model, which can adaptively improve the important channel characteristics and detection precision compared to model 1. Model 4 demonstrates significant improvements in all metrics compared to the original YOLOv5 when all three improvement methods are used simultaneously; AP reaches 98.76%, an improvement of 2.49%, recall reaches 96.18%, an improvement of 2.27%, and detection speed reaches 82 frames, an improvement of 17 frames. Compared to the various combinations of improvement methods for Model 1, 2, and 3, Model 4 exhibits a significant rise in recall and average precision. Clearly, these three improved methods complement one another and play an important role in the detection of small targets.

In order to demonstrate the detection performance of the improved YOLOv5-QCB model presented in this paper, the algorithm is compared to four widely used target detection algorithms, SSD [23], YOLOv3 [24], YOLOv4 [25], and YOLOv5s, using the same datasets. The experimental outcomes are presented in Table 5. Table 5 demonstrates that the improved model YOLOv5-QCB performs well for detecting bubbles in the transparent layer of quartz crucibles in terms of all term performance indicators. Although the YOLOv5-QCB proposed in this paper has slightly lower precision than SSD, it has significant advantages in recall, average precision, and model size. Compared to the classical algorithms of YOLOv3, YOLOv4, and YOLOv5s, which are more balanced in speed and precision, YOLOv5-QCB not only has the smallest model weight, but also the highest recognition precision and border regression capability, as well as superior overall performance.

**Figure 12:**
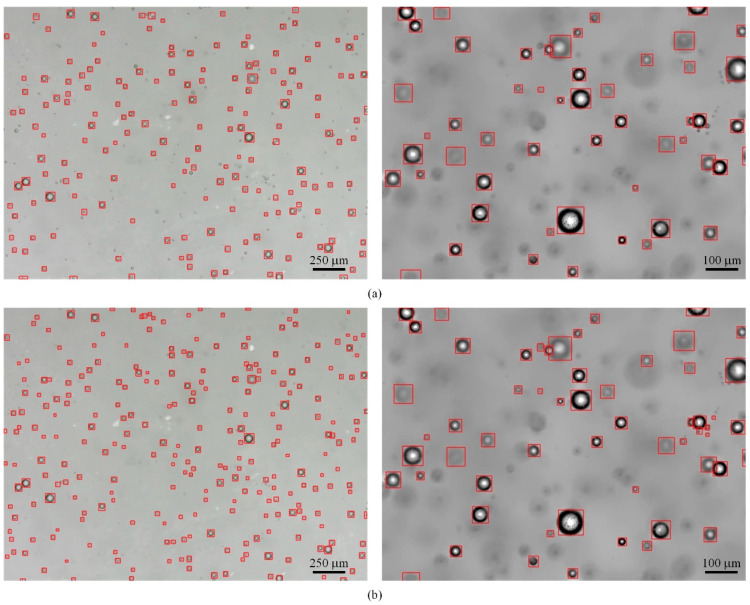
Comparison of visual graphs of test results. (**a**) The results of YOLOv5s testing; (**b**) The results of YOLO-QCB testing.

To evaluate the effects of the original YOLOv5s and the improved YOLOv5-QCB on the detection results, images of bubbles of various sizes and densities were tested. The results of the visualization are shown in Figure 12. It can be seen that YOLOv5-QCB is optimal for bubble target detection in different scenes. YOLOv5-QCB can detect missed targets in YOLOv5s under condition of low light, low contrast between bubble image outline and background, and high noise interference, demonstrating its superior dense multi-target detection and small target detection capability.

When the camera is equipped with a 200× magnification prime focus microscopic lens, the resolution of the original image is 640 pixels × 480 pixels. Under this configuration, the actual area corresponding to the image is 1520 µm × 1140 µm, and the actual size corresponding to each pixel is 2.375 µm × 2.375 µm. In this paper, through the relationship between the pixel area and the actual corresponding area, the bubble diameter can be obtained from the detection box in Figure 12 after image processing. In the left figure, the maximum diameter of bubbles is 89.063 µm, and the average diameter is 47.025 µm. In the figure on the right, the maximum value of the bubble is 97.375 µm, and the average diameter is 51.775 µm.

### 4.5. Tracking Algorithm Comparison

The experiments in this section aim to analyze the performance of the enhanced YOLOv5-QCB + SORT algorithm by testing it on a variety of video sequences to determine the algorithm’s applicability and validity in various environments. Moreover, based on the relationship between the tracker and detector, the algorithm of this paper is compared to other fusion algorithms to demonstrate its superiority.

Table 6 displays the results of the experiment; four video sequences were selected, with varying target densities and sizes. The proposed algorithm performs most accurately in video sequence 3, counting dense small bubbles with 97.3% accuracy. For video sequence 1 and 2 counts, an accuracy of 94.4% and 96.2% is achieved, respectively. Even though video 4 contains bubbles that are more difficult to detect, the counting accuracy can still reach 91.7%. In conclusion, the proposed algorithm can accurately determine the number of bubbles in a video by detecting and tracking bubble targets in a variety of shooting environments.

Table 7 compares the experimental outcomes of the algorithms proposed in this paper to those of other algorithms. The improved YOLOv5-QCB and SORT algorithms presented in this paper are the most effective methods for counting the number of bubbles in a video due to their high accuracy and detection speed. In terms of counting accuracy, the YOLOv5s + SORT, YOLOv4 + SORT, YOLOv3 + SORT, and SSD + SORT algorithms all perform poorly and are unable to meet the requirement for counting accuracy. This is primarily due to the fact that the tracking quality is highly dependent on the detector’s performance, i.e., the detector’s performance has an outsized impact on the counting accuracy. Due to the fact that the YOLOv4, YOLOv3, and SSD detectors have not been improved, it is difficult to detect small bubbles in videos, resulting in poor tracking results and low counting accuracy. In this paper, the best performance of the YOLOv5-QCB detector and the SORT tracker has been achieved by employing a series of enhancement strategies to improve the recognition of small targets.

After using this algorithm to complete frame-by-frame detection for an image with an actual area of 1520 µm × 1140 µm, the image information with the diameter of detected bubbles greater than 100 µm and the number of bubbles in a single frame greater than 100 is saved. This allows for convenient follow-up information queries and effectively improves the detection efficiency of quartz crucible.

## 5. Conclusions

To improve the detection accuracy of the detector for small bubble targets and to count the number of bubbles in the crucible space of the video, a YOLOv5-QCB combined with SORT counting method is proposed in this paper. First, to address the issue of loss of detailed information in the original YOLOv5 model, it is experimentally confirmed that sufficient local detailed features can be retained after the 32-fold down-sampling layers are removed. Then, in the neck network, the feature map perceptual field is expanded using dilated convolution to achieve the fusion of local detailed features with global semantic features. Moreover, prior to head detection, the ECA-Net mechanism is used to improve the representation of vital channel characteristics. These strategies collectively improve the detection performance of small bubbles and drastically reduce the parameters and computational effort. Lastly, the improved YOLOv5-QCB model is used for small bubble detection and is combined with the SORT tracking algorithm to count the number of intact bubbles in the spatial videos of quartz crucibles captured by microscopy, enabling a simple, efficient, and practical counting method. The method provides technical support for the implementation of industrial inspection automation.

## Figures and Tables

**Table 1:** Anchor box size allocation.

Feature Map Scale	Anchor Box		
80 × 80	(8, 8)	(13, 13)	(17, 17)
40 × 40	(22, 22)	(28, 28)	(38, 39)

**Table 2:** Training and test set information.

Datasets	Category Name	Number of Images	Number of Targets
Training Set	QCB	400	4878
Validation Set	QCB	100	1339

**Table 3:** Comparative analysis of several depth feature fusion structures.

Model	Precision (%)	Recall (%)	AP (%)	Weight (MB)	Speed (fps)
A	94.77	93.91	96.27	13.70	65
B	94.73	93.06	95.51	12.50	83
C	94.66	94.69	97.31	1.16	101
D	94.86	95.40	97.66	3.85	89

**Table 4:** Comparative ablation experiments using several improved method combinations.

Model	Shallow Network Structure	Dilated Convolution	ECA-Net	Precision (%)	Recall (%)	AP (%)	Weight (MB)	Speed (fps)
YOLOv5s				94.77	93.91	96.27	13.70	65
1	√			94.86	95.40	97.66	3.85	89
2	√	√		95.35	95.96	98.38	6.11	85
3	√		√	95.90	94.69	98.06	3.85	87
4	√	√	√	95.03	96.18	98.76	6.11	82

**Table 5:** Comparison of the performance of several detection algorithms.

Model	Precision (%)	Recall (%)	AP (%)	Weight (MB)	Speed (fps)
SSD	96.57	65.79	86.09	90.60	13
YOLOv3	88.24	89.31	92.35	235.00	14
YOLOv4	91.54	91.93	95.77	244.00	11
YOLOv5s	94.77	93.91	96.27	13.70	65
YOLOv5-QCB	95.03	96.18	98.76	6.11	82

**Table 6:** Results of testing the proposed algorithm on several video sequences.

Video	Detect Number	Actual Number	Accuracy (%)	Speed (fps)
Video1	34	36	94.4	49
Video2	50	52	96.2	45
Video3	177	182	97.3	31
Video4	33	36	91.7	42

**Table 7:** Comparison of various detection models in conjunction with the SORT algorithm.

Model	Detect Number	Actual Number	Accuracy (%)	Speed (fps)
SSD + SORT	212	306	69.3	11
YOLOv3 + SORT	251	306	82.0	12
YOLOv4 + SORT	254	306	83.0	10
YOLOv5s + SORT	267	306	87.3	37
YOLOv5-QCB + SORT	294	306	96.1	41

## Data Availability

Data available on request from the corresponding author.
